# Resveratrol induces autophagy by directly inhibiting mTOR through ATP competition

**DOI:** 10.1038/srep21772

**Published:** 2016-02-23

**Authors:** Dohyun Park, Heeyoon Jeong, Mi Nam Lee, Ara Koh, Ohman Kwon, Yong Ryoul Yang, Jungeun Noh, Pann-Ghill Suh, Hwangseo Park, Sung Ho Ryu

**Affiliations:** 1 Department of Life Sciences, Pohang University of Science and Technology, Pohang 790-784, Republic of Korea; 2 School of Interdisciplinary Bioscience and Bioengineering, Pohang University of Science and Technology, Pohang 790-784, Republic of Korea; 3 School of Life Sciences, Ulsan National Institute of Science and Technology, Ulsan 689-798, Republic of Korea; 4 Department of Bioscience and Biotechnology, Sejong University, 98 Kunja-Dong, Kwangjin-Ku, Seoul 143-747, Republic of Korea

## Abstract

Resveratrol (RSV) is a natural polyphenol that has a beneficial effect on health, and resveratrol-induced autophagy has been suggested to be a key process in mediating many beneficial effects of resveratrol, such as reduction of inflammation and induction of cancer cell death. Although various resveratrol targets have been suggested, the molecule that mediates resveratrol-induced autophagy remains unknown. Here, we demonstrate that resveratrol induces autophagy by directly inhibiting the mTOR-ULK1 pathway. We found that inhibition of mTOR activity and presence of ULK1 are required for autophagy induction by resveratrol. In line with this mTOR dependency, we found that resveratrol suppresses the viability of MCF7 cells but not of SW620 cells, which are mTOR inhibitor sensitive and insensitive cancer cells, respectively. We also found that resveratrol-induced cancer cell suppression occurred ULK1 dependently. For the mechanism of action of resveratrol on mTOR inhibition, we demonstrate that resveratrol directly inhibits mTOR. We found that resveratrol inhibits mTOR by docking onto the ATP-binding pocket of mTOR (i.e., it competes with ATP). We propose mTOR as a novel direct target of resveratrol, and inhibition of mTOR is necessary for autophagy induction.

Resveratrol (3,40,5-trihydroxy-trans-stilbene) is a natural polyphenolic compound found in the roots of plants and in edible fruits including berries and grapes. Administration of resveratrol is thought to elicit beneficial effects, including alleviation of inflammation and tumor cell death in applied cells and organisms^1^. Autophagy is a cellular process that removes damaged organelles or cellular constituents and provides energy under starvation conditions or repairs damage under stressed conditions. The autophagic process is of great interest because of its high association with various diseases, including cancer, neurodegenerative diseases, myopathy, and cardiac disease. Aberrant regulation of autophagy has been observed in various diseases, and activation of autophagy is known to alleviate symptoms and perhaps even cure these diseases^2^,^3^. Recently, resveratrol was suggested to induce autophagy, and this process is responsible for the beneficial effects of resveratrol, including reduction of inflammation, induction of tumor cell death, and protection against oxidative damage^4^. Several groups have attempted to explain the mechanism by which resveratrol induces autophagy and have suggested the mediator of this process^5^,^6^,^7^. Nonetheless, evidence directly linking resveratrol to autophagy is still lacking.

mTOR (the mammalian or mechanistic target of rapamycin), a Ser/Thr kinase present in cells, functions within two distinct complexes: mTORC1 (mTOR complex 1) and mTORC2 (mTOR complex 2). These complexes share mTOR as a kinase subunit, but contain different adaptors and scaffolds for distinct functions and regulatory mechanisms. mTORC1 controls cell growth and proliferation through the regulation of various processes and promotes translation through the phosphorylation of S6K and 4E-BP1. Lipid synthesis is also enhanced by active mTOR through the regulation of PPAR-γ, SREBP, and Lipin1^8^,^9^. In addition to these anabolic processes, mTORC1 also suppresses catabolism by inhibiting autophagy. Inhibition of mTORC1 is sufficient to induce autophagy, and nutrient-insensitive mTOR renders cells unresponsive to starvation-induced autophagy^10^. Active mTOR inhibits autophagy by suppressing the ULK1-ATG13-FIP200 complex, specifically through the inhibitory phosphorylation of ULK1^11^,^12^,^13^,^14^. Several studies have shown that resveratrol suppresses mTOR activity, which is expected to mediate resveratrol-induced autophagy. For the mechanism of inhibition, involvement of upstream regulators, including PI3K, AMPK, and SIRT1, has been suggested^5^,^15^,^16^. However, conflicting results between these reports for the requirement of above-mentioned regulators in resveratrol-induced mTOR suppression result in ambiguity.

Here, we demonstrate that resveratrol induces autophagy through the mTOR-ULK1 pathway. Additionally, we found that resveratrol induces death of the cancer cells known to be sensitive to mTOR inhibition in ULK1 dependent manner. With respect to the mechanism by which resveratrol reduces mTOR activity, we demonstrated that resveratrol directly inhibits mTOR in an ATP-competitive manner. Through this study we suggest that mTOR is a distinct and direct target of resveratrol, and inhibition of mTOR is necessary for resveratrol-induced autophagy.

## Results

### Resveratrol induces autophagy through mTOR inhibition

**Figure 1:**
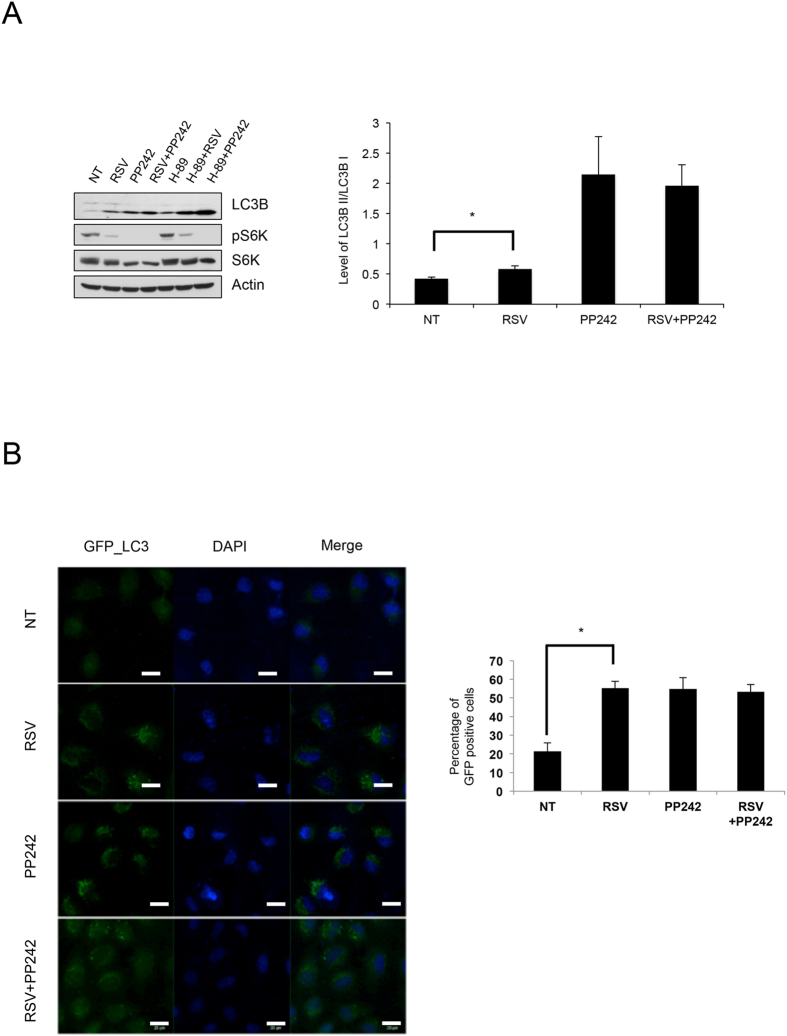
Resveratrol induces autophagy through mTOR inhibition. (**A**) Autophagy induction was analyzed by measuring accumulation level of LC3-II. Resveratrol (100 μM), PP242 (1.25 μM), H-89 (10 μM), or a combination of two different chemicals was administered to HEK293 cells for 4 h. *P < 0.05 n = 3. (**B**) LC3 puncta formation induced by resveratrol (50 μM), PP242 (1.25 μM), or a combination of these chemicals was examined in HeLa cells stably expressing GFP-LC3 for 2 h. Scale bars in fluorescent pictures represent 20 μm

In order to understand resveratrol-induced autophagy, we examined the effect of resveratrol on autophagy in GFP-LC3 expressing HeLa cells. Resveratrol treatment induces autophagy, as evidenced by the accumulation of LC3B-II and LC3 puncta formation^14^ (supplementary Fig. S1). Along with this result, treatment with mTOR kinase inhibitor pp242 demonstrated that mTORC1 activity is inversely correlated with the level of autophagy (supplementary Fig. S1A). This result implicates the possible involvement of mTORC1 in resveratrol-induced autophagy. To test the position of mTOR in resveratrol-induced autophagy, we used various drugs that induce autophagy. We found that combinatory treatment with resveratrol and PP242 did not show any additive effect (Fig. 1A,B), indicating that resveratrol acts via the same pathway as PP242. Decreased cyclic-AMP (cAMP) levels induce autophagy in an mTOR-independent manner^17^. Because reduction of cAMP induces autophagy through inhibition of PKA^18^, we utilized H-89, a PKA inhibitor, to induce mTOR-independent autophagy. As expected, treatment with H-89 increased autophagy without reduction of mTORC1 activity. Additionally, unlike the resveratrol-PP242 combination, co-treatment with H-89 and resveratrol or H-89 and PP242 had an additive effect on the accumulation of LC3B-II (Fig. 1A). This result indicates that H-89 induces autophagy through an mTOR-independent pathway, and resveratrol and H-89 use different pathways to induce autophagy. Taken together, these combinatory drug treatment experiments indicate that resveratrol induces autophagy at the level as same as mTOR inhibition.

### ULK1 is required for resveratrol-induced autophagy

**Figure 2:**
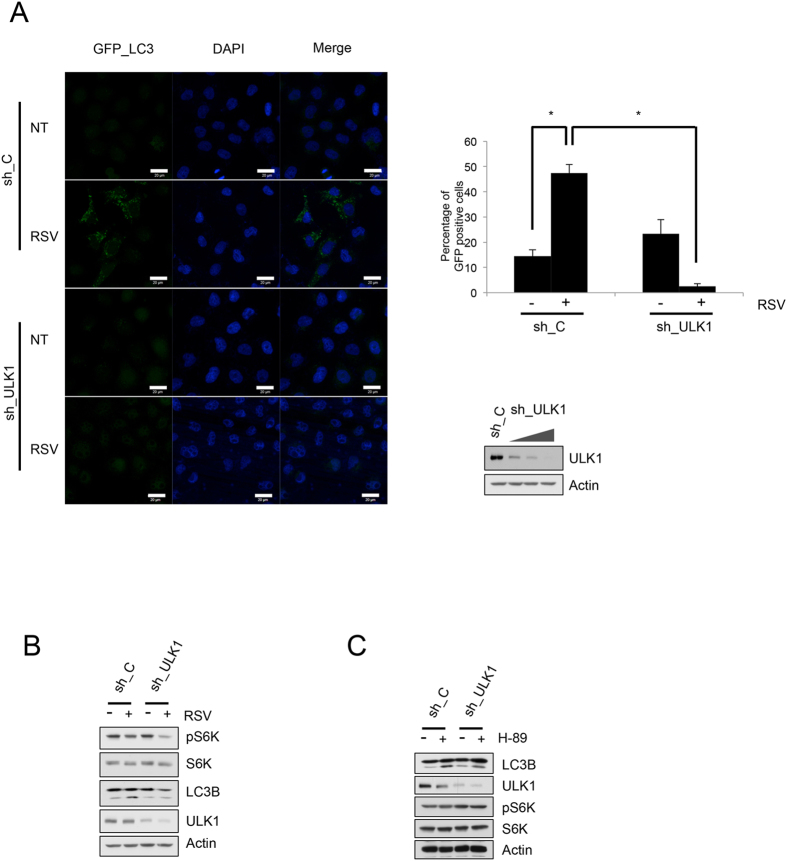
ULK1 is required for resveratrol-induced autophagy. (**A**) Autophagy induction by resveratrol (50 μM, 2 h) in the presence or absence of ULK1. Lenti virus encoding vector or shULK1 was introduced into GFP-LC3 expressing HeLa cells. (**B**) Autophagy induction by resveratrol (50 μM, 2 h)in the presence or absence of ULK1. cDNA encoding vector or shULK1 was transfected into HEK293 cells. Scale bars in fluorescent pictures represent 20 μm. (**C**) Autophagy induction by H-89 (10 μM, 2 h) in the presence or absence of ULK1. cDNA encoding vector or shULK1 was transfected into HEK293 cells.

mTOR regulates autophagy through inhibitory phosphorylation of ULK1 and inhibition of mTOR decreases the inhibitory phosphorylation level of ULK1 and increases autophagy^13^,^14^. Therefore, to confirm the involvement of mTOR in resveratrol-induced autophagy and to examine the ULK1 dependency, we analyzed the level of autophagy induced by resveratrol in the presence or absence of ULK1. ULK1 knockdown using shRNA abolished resveratrol-induced autophagy, indicating that ULK1 is required for resveratrol-induced autophagy (Fig. 2A,B). However, ULK1 knockdown did not abolish autophagy induced by H-89 treatment (Fig. 2C), again confirming that the mTOR-regulated autophagy is independent of the cAMP-PKA pathway. Together, we concluded that resveratrol induces autophagy through the mTOR-ULK1 pathway, which includes inhibition of mTOR.

### Resveratrol reduces viability of mTOR inhibition sensitive cancer cells in ULK1 dependent manner

**Figure 3:**
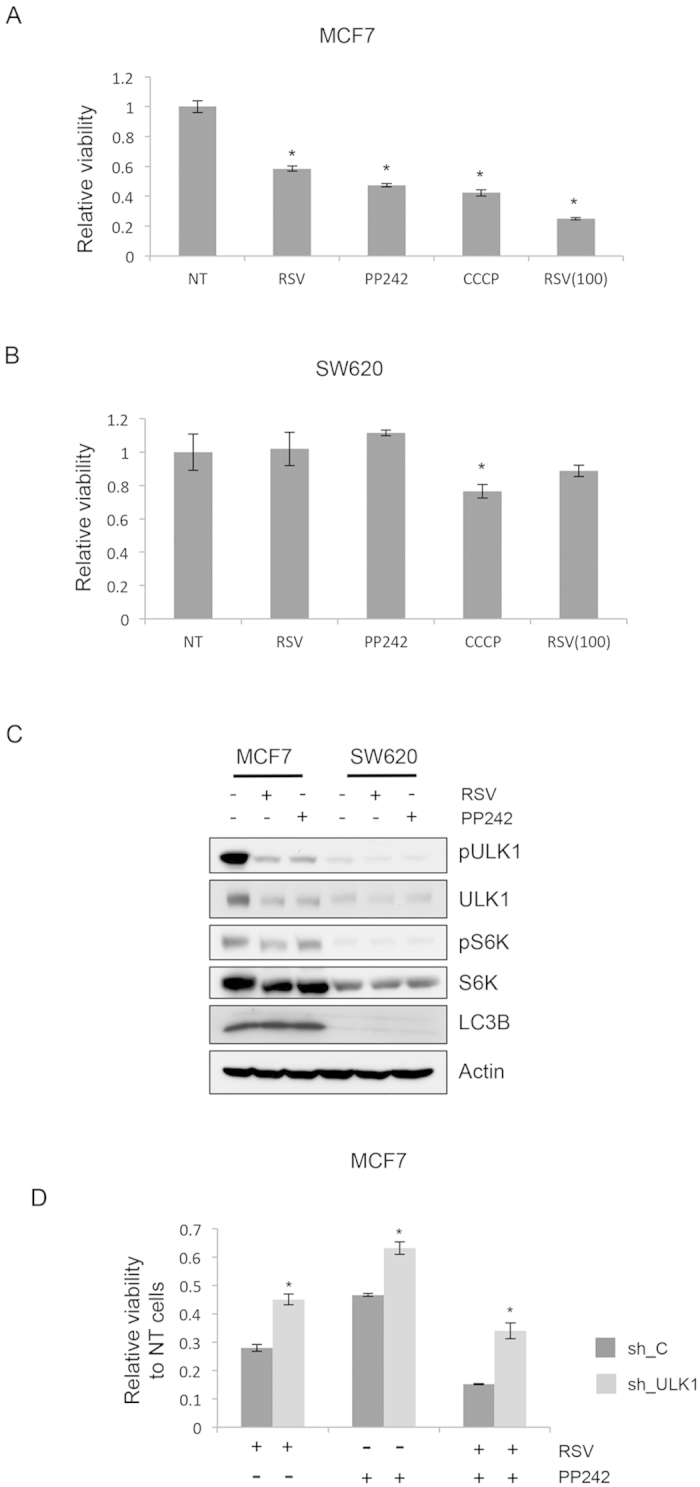
Resveratrol reduces viability of mTOR inhibition sensitive cancer cells. (**A**) MCF7 Cells were treated with resveratrol (50 or 100 μM) or PP242 (500 nM) or CCCP (10 μM) for 48 h followed by cell viability measurement by MTT assay. (**B**) Chemical treatment and viability measurement for SW620 cells were conducted same as MCF7 cells. (**C**) Intra-cellular signaling was examined in MCF7 cells and SW620 cells after 4 h treatment of resveratrol (50 μM) or PP242 (500 nM). n = 3. (**D**) MCF7 cells were infected with lenti virus containing vector or shULK1. After 48 h infection chemical treatment was conducted for another 48 h followed by viability measurement. The Y-axis of the graph indicates the levels relative to those of untreated negative control samples.

As previous studies showed, resveratrol-induced autophagy suppresses cancer *in vitro* and *in vivo*^4^,^6^,^19^. Therefore, we examined whether resveratrol-induced suppression of cancer progression occurred through mTOR and inhibition of ULK1. To examine mTOR dependency on resveratrol induced-cancer cell suppression, we utilized two different cell lines that were previously reported mTOR inhibition sensitive and insensitive, respectively^20^. MCF7, a breast cancer cell that has constitutive PI3K activation and higher mTOR activity so that it is sensitive to mTOR inhibitor, showed very sensitive response to resveratrol treatment (Fig. 3A). However, neither PP242 nor resveratrol reduced the viability of Ras transformed colorectal cancer cells, SW620 (Fig. 3B). This result indicates that resveratrol effect on cancer cell viability is largely dependent on impact of mTOR, which varies in cancer cell types. To examine whether the effect of resveratrol on cellular viability is reflected in intra-cellular signaling, we measured mTOR activity in MCF7 and SW620 cells upon resveratrol and PP242 treatment. The basal level of mTOR activity and responsiveness to resveratrol and PP242 were significantly lower in SW620 cells compared to MCF7 cells, further demonstrating that the modulation of mTOR activity is involved in the physiological effects of resveratrol (Fig. 3C). Next, we examined whether ULK1 is involved in resveratrol-induced suppression of cancer cell viability. Like knockdown of ULK1 blunted the autophagy induction by resveratrol, reduction of viability of MCF7 cells was partially restored by ULK1 knockdown (Fig. 3D). This restoration of viability was also observed in PP242 induced cancer cell suppression (Fig. 3D). These cancer cell specific and ULK1 dependent effect of resveratrol further supports the idea that resveratrol-induced cellular behavior alterations occurred through mTOR-ULK1 pathway.

### mTOR-associated DEPTOR level is not changed by resveratrol

DEPTOR is a negative regulator of both mTORC1 and mTORC2. Liu *et al.* showed that resveratrol increased the interaction affinity between mTOR and DEPTOR and suggested that DEPTOR is required for the suppression of mTOR activity upon resveratrol treatment^21^. To examine the involvement of DEPTOR, we conducted a pull-down assay of FLAG-tagged mTOR under various conditions, including resveratrol treatment. Rapamycin was used as a positive control for chemically-induced complex dissociation. Unexpectedly, in this condition, both the 10 min and 60 min resveratrol treatments did not increase the affinity between mTOR and DEPTOR although resveratrol clearly decreased mTOR activity. Treatment with rapamycin led to the detachment of Raptor from mTOR (supplementary Fig. S2).

### Resveratrol inhibits mTOR kinase activity in cell-free systems

**Figure 4:**
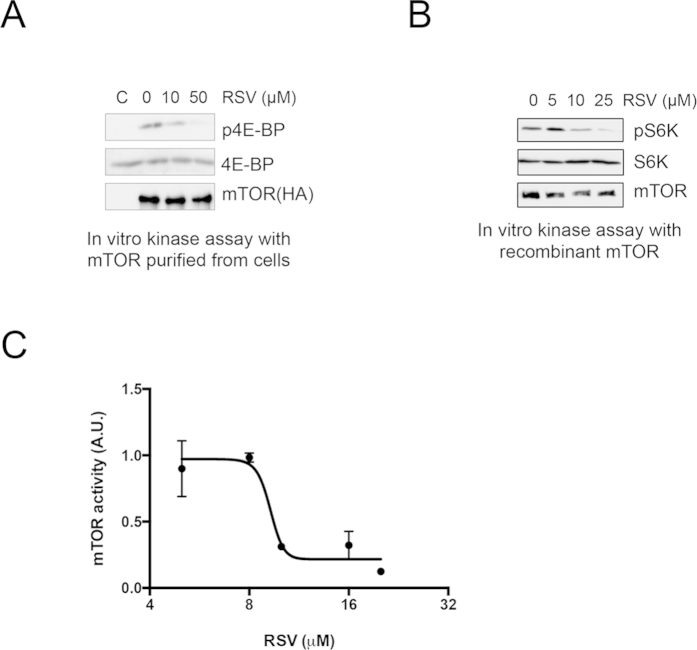
Resveratrol inhibits mTOR kinase activity in cell-free systems. (**A**) An *in vitro* mTOR kinase assay was performed with purified mTOR from HEK293 cells. HA-tagged mTOR was introduced into cells and immunoprecipitated with the anti-HA antibody. (**B**) An *in vitro* mTOR kinase assay was performed using GST-tagged recombinant mTOR. (**C**) Inhibition curve of mTOR activity with the indicated concentration of resveratrol. An *in vitro* kinase assay was performed as in B. n = 3.

Next, we examined whether resveratrol directly suppresses mTOR activity. To address this question, we performed an *in vitro* kinase assay. We observed that resveratrol dose-dependently decreased the phosphorylation level of 4E-BP1 (Fig. 4A). In addition to the experiments with full-length mTOR, we used a recombinant fragment mTOR that some regulatory domains are deleted, but still contains the intact kinase domain for a kinase assay. Notably, resveratrol could decrease the phosphorylation of S6K, a substrate of mTOR (Fig. 4B), similar to the results of immunoprecipitated full-length mTOR (Fig. 4A). Additionally, we found that resveratrol has an IC_50_ value of ~10 μM against mTOR kinase activity (Fig. 4C). Taken together, resveratrol inhibits mTOR kinase activity in cell-free systems.

### Computational simulation of the interaction between mTOR and resveratrol

**Figure 5:**
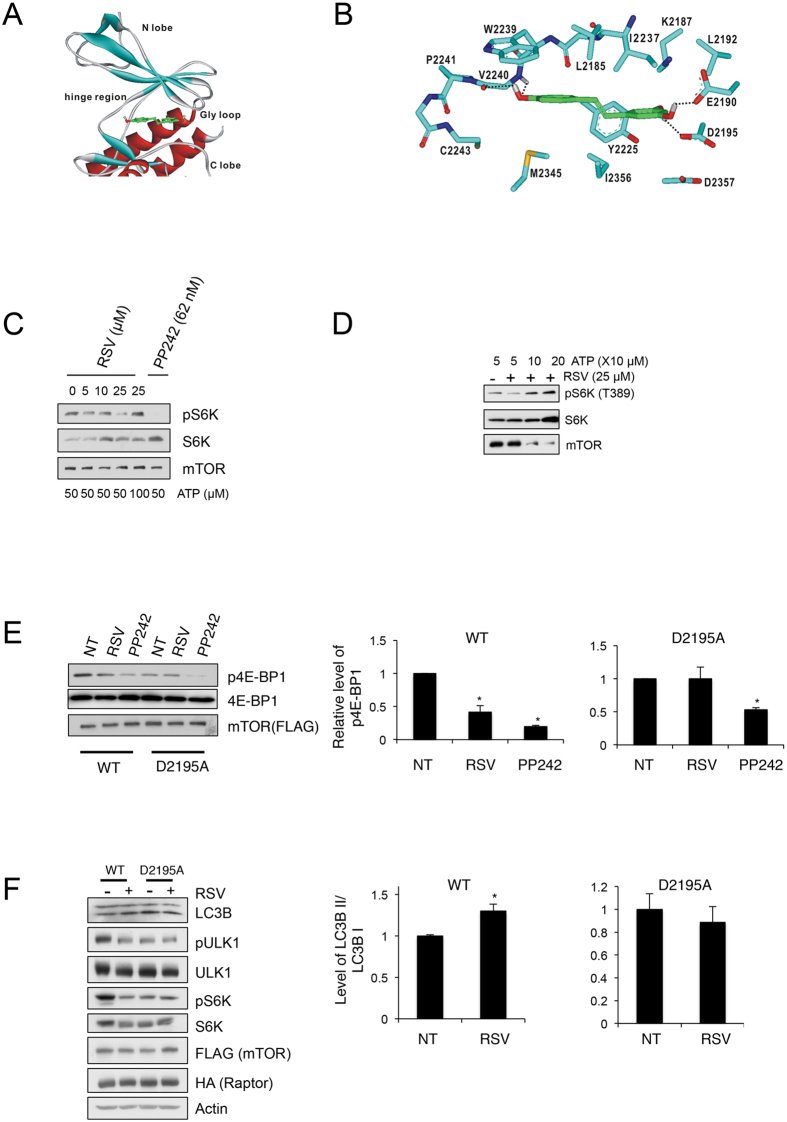
Resveratrol inhibits mTOR through ATP competition. (**A**) Docking pose of resveratrol in the ATP-binding site of mTOR. (**B**) Detailed binding mode of resveratrol in the ATP-binding site of mTOR. The carbon atoms of resveratrol and mTOR are shown in green and cyan, respectively. Hydrogen bonds are indicated with dotted lines. (**C**) Suppression of activity by resveratrol was dependent on the level of ATP. An *in vitro* kinase assay was performed with the indicated amounts of ATP and resveratrol. mTOR kinase inhibitor PP242 was used as a positive control. (**D**) *In vitro* kinase was performed with increased amount of ATP. (**E**) *In vitro* kinase was performed with WT mTOR and D2195A mTOR *P < 0.05 n = 3. (**F**) WT mTOR or D2195A mTOR, and HA-Raptor were transfected into HEK293T cells. After 24 h post transfection, Resveratrol (50 μM) was administered to HEK293 cells for 4 h to measure the accumulation of LC3B-II. *P < 0.05 n = 3.

To assess the mode of action for inhibiting the kinase activity of mTOR, we carried out docking simulations of resveratrol in the ATP-binding site of mTOR for determining the lowest-energy binding mode of resveratrol (Fig. 5A). Resveratrol appears to be stabilized in the binding pocket formed by the Gly loop, hinge region of the ATP-binding site, and the interface residues of N- and C-terminal domains. To examine the possibility of allosteric inhibition of mTOR by resveratrol, we performed additional docking simulations with extended 3D grid maps that include the whole kinase domain. However, no peripheral binding site was found in which resveratrol could be stabilized with negative free energy of binding. It is thus expected that the micromolar-level inhibitory activity of resveratrol against mTOR stems from the specific binding in the ATP binding site.

We now address the detailed interactions responsible for stabilization of resveratrol in the ATP-binding pocket. The binding mode of resveratrol calculated from docking simulations is shown in Fig. 5B. We observed that the terminal phenolic group of resveratrol receives and donates a hydrogen bond from the backbone amidic nitrogen to the aminocarbonyl oxygen of V2240, respectively. This hydrogen bond seems to be important for the biochemical potency of resveratrol because the formation of hydrogen bonds with same region was also observed in the structures of mTOR complex with potent inhibitors^22^. In the assessed mTOR-resveratrol complex, two additional hydrogen bonds are established between the benzene-1,3-diol moiety and the side-chain carboxylate groups of E2190 and D2195. On the basis from docking simulations, it can be argued that the inhibitory activity of resveratrol can be attributed to the multiple hydrogen bonds.

### Resveratrol inhibits mTOR through ATP competition

To verify the simulation results, we performed an *in vitro* kinase assay with various concentrations of ATP. PP242, a kinase inhibitor of mTOR, was used as a positive control for mTOR inhibition. We found that resveratrol-induced mTOR inhibition was restored by the addition of ATP (Fig. 5C,D). To clarify whether resveratrol inhibits mTOR through direct interaction, we generated a resveratrol-resistant mTOR mutant. Based on the resolved mTOR-ATP structure^22^ and our computational simulation (Fig. 5A,B), Asp 2195 was deemed to be a resveratrol-binding residue that does not affect the mTOR-ATP interaction. Thereafter, we generated an mTOR mutant in which Asp 2195 was substituted with alanine (hereafter, referred to as D2195A) and performed an *in vitro* kinase assay. As expected, resveratrol inhibited wild-type (WT) mTOR but not D2195A mTOR. The basal activity level of the D2195A mutant was lower than that of WT mTOR, possibly due to the fact that the mutated residue resides in the kinase domain of the protein. We observed that PP242 also inhibited DA mTOR, albeit the inhibitory potency was less than WT mTOR (Fig. 5E). Although Asp 2195 was suggested as a residue for the interaction between mTOR and PP242, other residues also participate the interaction between them and not all residues are shared between PP242 and resveratrol for mTOR binding. We speculate that other residues but not D2105 may be essential for the PP242 binding to mTOR and that is why D2195A mTOR was not completely resistant to PP242. To examine whether the resveratrol resistant mTOR blocks the resveratrol-induced autophagy, we measured the autophagy levels upon resveratrol treatment in the WT or D2195A mTOR along with Raptor transfected cells. We found that resveratrol induced autophagy in the WT mTOR transfected cells, but not in the D2109A mTOR transfected cells (Fig. 5F). Taken together, the results from the experiments using resveratrol resistant mTOR suggest that resveratrol induces autophagy by directly inhibits mTOR through ATP competition.

## Discussion

We demonstrated that mTOR is the direct target of resveratrol. This claim is supported by the observation that mTOR activity is inhibited by addition of resveratrol *in vitro*. Additionally, this study provides a mechanistic explanation for the beneficial effects of resveratrol, which is mTOR-dependent autophagy induction and reduction of cancer cell viability. These findings also increase the possibility of application of resveratrol to maladies that are highly associated with active mTOR, such as neurodegenerative diseases and diabetes.

Because of its beneficial effects, many researchers have attempted to identify the molecular target of resveratrol. SIRT1, a NAD^+^-dependent deacetylase, was initially identified as the direct target of resveratrol by *in vitro* screening^23^. Additionally, other studies showed that the administration of resveratrol to mice and cells decreased the acetylation level of SIRT1 targets^24^. The similarity in the functional outcome of SIRT1 activation and resveratrol administration suggested that SIRT1 can be a direct physiological target of resveratrol. However, several later studies reported that resveratrol did not activate SIRT1 in *in vitro* assays when native peptides, not fluorophore-tagged peptides (used in the original *in vitro* assay), were used as substrates. These results suggested that SIRT1 may not be the direct target of resveratrol, although resveratrol clearly activates SIRT1 *in vivo*^25^,^26^,^27^,^28^. Resveratrol was reported to activate AMPK (AMP-activated kinase) and additional studies have suggested that AMPK activation mediates the beneficial effects of resveratrol administration^29^,^30^,^31^,^32^. Although resveratrol activates AMPK, AMPK is unlikely to be a direct target of resveratrol^33^,^34^. After a long search for a direct target of resveratrol, Park *et al.* suggested PDEs (phosphodiesterases) as a direct target of resveratrol. In their report, they found that resveratrol inhibits PDEs through competition with cAMP (cyclic AMP), a substrate of PDE. They also observed that inhibition of PDE is enough to mimic the effects of resveratrol administration in mice, such as AMPK activation, SIRT1 inhibition, and enhancement of metabolic features^35^. Since the above-mentioned molecules are the known upstream regulators of mTORC1, inhibition of mTOR by resveratrol was thought to be due to these upstream regulators. However, we found that mTOR inhibition by resveratrol is independent of AMPK, SIRT1, PDE, and PI3K (data not shown).

Resveratrol was initially identified as a SIRT1 activator because of its structural similarity to quercetin^23^. Considering that quercetin is known to inhibit a broad spectrum of kinases in an ATP-competitive manner and that resveratrol also inhibits several kinases^36^, increased the possibility of ATP competitive mode of action of resveratrol in mTOR inhibition. To test the possibility that resveratrol directly binds in the ATP-binding site of mTOR, we carried out docking simulations between mTOR and resveratrol (Fig. 5). Detailed binding mode analysis of resveratrol implied that its binding in the ATP-binding site of mTOR could be facilitated by the establishment of multiple hydrogen bonds with the backbone amide groups in the hinge region and the side chains of E2190 and D2195. Simultaneously, hydrophobic interactions with the nonpolar residues in the ATP-binding pocket were also found to be a significant binding force that stabilizes the mTOR-resveratrol complex. These computational results may serve as persuasive evidence for competitive inhibition of mTOR by resveratrol. An analysis of mTOR activity using a putative resveratrol binding-defective mutant verified this result by showing that resveratrol suppressed WT mTOR but not D2195A mTOR (Fig. 5E). The Asp 2195 residue is also involved in mTOR-PP242 binding^22^. However, PP242 inhibited the D2195A mutant, though its potency was reduced compared to WT mTOR. This result indicates that Asp 2195 is a key residue for mTOR-resveratrol binding.

Resveratrol was reported to directly inhibit various enzymes as demonstrated by *in vitro* activity assays. Among these enzymes, PI3K and PKC showed ATP-dependent inhibition by resveratrol^37^,^38^. However, the studies provided no evidence for the link between the inhibition of these molecules and the beneficial effects of resveratrol administration. Further studies are needed to determine the position of physiological targets of resveratrol. In addition to kinases, PDE, which uses cAMP as a substrate, is also inhibited by resveratrol in a substrate-competitive manner. Notably, mTOR had the lowest IC_50_ value for resveratrol compared to PI3K, PKC, and PDE. Resveratrol, ATP, and cAMP are similar in structure, which suggested that resveratrol could compete with cAMP or ATP for single binding sites. Additionally, PP242 and quercetin, an mTOR kinase inhibitor and a natural polyphenol, respectively, are similar in structure.

We found that resveratrol showed a cancer-cell-type-selective effect on viability in that it suppressed only the viability of MC7 cells, but not that of SW620 cells (Fig. 3). However, we could not find any differences in the level of apoptosis between MCF7 and SW620 cells, which may indicate the involvement of other cellular processes. Notably, Colin *et al.* suggested acquired resistance to resveratrol in SW620 cells, which is dependent on the DNA-damage response^39^. Therefore, it remains to be determined whether mTOR and the DNA-damage response are associated with the determination of resveratrol resistance in SW620 cells.

Liu *et al.* showed that resveratrol suppresses mTOR activity by enhancing the affinity between mTOR and DEPTOR, a negative regulator of mTOR. However, we observed no changes in the level of mTOR-interacting DEPTOR (supplementary Fig. S2). This discrepancy may be explained by the differences in experimental conditions. They used a pull-down assay of endogenous mTOR from C2C12, while we immunoprecipitated FLAG-mTOR from HEK293 cells. Additionally, active mTOR was reported to promote the degradation of DEPTOR^12^. Thus, the increase in DEPTOR affinity may be due to the increased level of total DEPTOR proteins within cells due to resveratrol. Altogether, our study suggests that mTOR is a high-affinity direct target of resveratrol.

Resveratrol induces autophagy in various cell lines and in model organisms, such as *Caenorhabditis elegans* and mice^5^,^6^,^7^. In these reports, authors showed that autophagy induction is necessary for cancer cell death and alleviation of inflammation, and it may even extend the lifespan. Some studies showed decreased mTOR activity along with autophagy induction with resveratrol treatment, but there is no direct evidence suggesting that mTOR is a mediator of autophagy induction by resveratrol^5^,^15^,^16^,^40^. In this study, using a combinatory chemical treatment and ULK1 knockdown, we clearly demonstrated that the mTOR-ULK1 pathway is necessary for autophagy induction by resveratrol. In addition to resveratrol, other natural polyphenols, including quercetin and rottlerin also induce autophagy, possibly through mTOR^4^. Further studies are necessary to examine the involvement of mTOR in autophagy induction by other natural polyphenols. Inhibition of mTOR has been shown to be a good strategy in the treatment of various illnesses, such as cancer, neurodegenerative diseases, and diabetes^41^. This study will provide a basis for the identification of unknown mTOR-based effects of resveratrol.

## Methods

### Cell culture

GFP_LC3 HeLa was generated as previously described^42^. The above-mentioned cells and HEK293 cells were maintained with DMEM (Dulbecco’s modified Eagle’s medium) complemented with 10% fetal bovine serum (FBS, Lonza). To measure mTOR activity upon resveratrol treatment, cells were pretreated with 50 μM resveratrol (Sigma-Aldrich) for 30 min before stimulation with insulin or amino acids. Amino acid or serum fasting was conducted by maintaining cells with HBSS (Hank’s balanced salt solution; Invitrogen) supplemented with 10% dialyzed FBS (Invitrogen) or DMEM only, respectively. Before the resveratrol treatment, 10 μM H-89 (Biomol) was add for 1 h. For autophagy formation analysis using Western blotting, 10 μM of resveratrol was treated into cells in the presence or absence of 10 μM of Bafilomycin A1 (Enzo) for 2 h and cells were subjected to SDS-PAGE and Western blotting.

### MTT assay

Cells were seeded onto 96-well plates, which were placed in an incubator overnight to allow for attachment and recovery. In brief, cells were treated with 100 μM resveratrol for 48 h and MTT was then dissolved to a concentration of 5 mg/ml in warm assay medium. A total of 20 μl MTT solution was transferred to each well to yield a final volume of 120 μl/well. Plates were incubated for 4 h at 37 °C in 5% CO_2_. Following incubation, supernatants were removed and 100 μl DMSO was added. Dissolved precipitates were measured with plated reader with wavelength at 540 nm.

### *In vitro* kinase assay and analysis of enzyme kinetics

mTORC1 kinase assay was performed as described previously, with some modifications^43^. Briefly, immunoprecipitated HA-mTOR (kindly provided by Dr. Sabaini, MIT) was washed three times and incubated with kinase assay buffer (20 mM HEPES, 50 mM KCl, 10 mM MgCl_2_), 200 ng of 4E-BP1 (Stratagene) and the indicated amount of resveratrol for 10 min on ice. The reaction was started with the addition of 50 or 100 μM ATP and was conducted at 30 °C for 10 min. The reaction was stopped by the addition of 5x sample buffer. For a kinase assay using recombinant mTOR, GST-mTOR (Life Technologies) was used and GST-S6K, which is generated by PCR amplification of kinase dead fragment S6K (322 to C-term) and subcloning into pGEX-4T1 vector was purified from E.coli and used as a substrate. An innhibitory curve was drawn using Sigma Plot.

### Immunoprecipitation and Western blotting

HA-mTOR cDNA, HA-Raptor cDNA (kindly provided by Dr. Sabaini, MIT), FLAG-mTOR cDNA (insertion of PCR product from HA-mTOR cDNA into pCMV vector) and FLAG-D2195A mTOR (generated by site directed mutagensis) were transfected with Lipofectamine (Invitrogen) according to the manufacturer’s instructions. Cells were lysed in buffer containing 40 mM HEPES, 120 mM NaCl, 50 mM NaF, 10 mM β-glycerophosphate, 1 mM EDTA, 10 mM sodium pyrophosphate, 1 mM PMSF, 1 μg/μl aprotinin, 2 μg/μl leupeptin and 0.3% CHAPS. Except for the IP samples, 1% TX-100 was used as a detergent. Then 700–1000 μg of protein was incubated with the respective antibody for 4 h followed by incubation with protein A-conjugated agarose beads. Antibody immobilized beads (Sigma-Aldrich) were used for FLAG-mTOR. Beads were washed three times with the lysis buffer and resuspended with the sample buffer. Prepared samples were subjected to SDS-PAGE (6–16% gradient) and transferred to NC membranes (Amersham Biosciences). The membranes were blocked with TTBS buffer containing 5% skim milk and incubated overnight at 4 °C with antibodies against ULK1 (Santa Cruz Biotechnology), Rictor (Bethyl Laboratories), actin (MP Biomedicals) and other proteins (Cell Signaling). The membranes were next washed with TTBS, incubated with HRP-conjugated secondary antibodies at room temperature for 1 h, and washed again with TTBS. Antibody-bound membranes were developed using ECL (Thermo Fisher).

### Microscopic analysis of autophagy formation

On day 1, HeLa cells stably expressing GFP_LC3 (kindly provided by Dr. Yu Li, Tsinghua University) were plated onto poly-lysine coated glass coverslips. On day 2, medium was changed with fresh medium containing DMSO or resveratrol at the indicated concentration for 2 h. The medium was then aspirated and cells were washed twice with PBS. Cells were fixed with 4% formaldehyde for 10 min at RT and stained with DAPI for 5 min at RT. Washing three times with PBS was conducted between each step. The coverslips were mounted on a slide glass and visualized using confocal microscopy (Carl Zeiss), and randomly captured images from various fields were used for autophagy formation analysis. GFP punctate positive cells were quantified as autophagy induced cells. The total number of cells was determined by measuring DAPI-positive cells.

### Lentiviral preparation and viral infection

Lentiviral shRNAs were prepared and transduced according to the Addgene protocol. Briefly, the pLKO empty vector or pLKO shULK1_8 (purchased from Addgene) was transfected with pMD2.G and psPAX2 (purchased from Addgene) using Lipofectamine according to the manufacturer’s instructions(Invitrogen). Media were collected at 24 h and 48 h after transfection. Collected media were centrifuged at 1200 rpm for 5 min and filtered with 0.45-μm filter. For virus infection, virus-containing media were diluted with growth medium and added to plated cells. Twenty-four hours after infection, the medium was replaced with fresh medium. Twenty-four hours after changing the medium, infected cells were used for the various experiments based on experimental purpose.

### Computational docking simulation

Three dimensional (3D) atomic coordinates were prepared from the X-ray crystal structure of mTOR in complex with the potent inhibitor, Torin2 (PDB code: 4JSX)^1^ as the receptor for docking simulations with resveratrol. Gasteiger-Marsilli atomic charges^2^ were assigned for all protein and ligand atoms to calculate the electrostatic interaction in the mTOR-resveratrol complex^44^. Docking simulations were then conducted with the AutoDock program^3^ to obtain the binding mode of resveratrol with respect to mTOR^45^. Of the 20 conformations of resveratrol generated in docking simulations, those clustered together have similar binding modes differing by less than 1.5 Å in positional root-mean-square deviation. The most stable binding configuration in the top-ranked cluster was selected for further analysis.

### Statistical Analyses

Microsoft Excel was used for statistical analysis. Data are presented as means ± SEM. Comparisons between two groups were made by unpaired two-tailed Student’s t-tests. p values < 0.05 were considered statistically significant.

## Additional Information

**How to cite this article**: Park, D. *et al.* Resveratrol induces autophagy by directly inhibiting mTOR through ATP competition. *Sci. Rep.* **6**, 21772; doi: 10.1038/srep21772 (2016).

## Supplementary Material

Supplementary Information
