# Supplementary material for: Resveratrol induces autophagy by directly inhibiting mTOR through ATP competition
Source: Sci Rep. 2016 Feb 23;6:21772. doi: 10.1038/srep21772 (PMC4763238; doi:10.1038/srep21772)

Resveratrol induces autophagy by directly inhibiting mTOR through ATP competition.

Authors: Dohyun Park[A], Heeyoon Jeong[A], Mi Nam Lee[A], Ara Koh[A], Ohman Kwon[B], Yong Ryoul Yang[C], Jungeun Noh[A], Pann-Ghill Suh[C], Hwangseo Park[D], Sung Ho Ryu[A,B,] *

A.

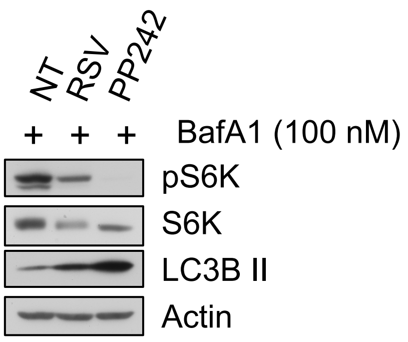

NT
RSV
PP242
+ + + BafA1 (100 nM)
pS6K
S6K
LC3B II
Actin

B.

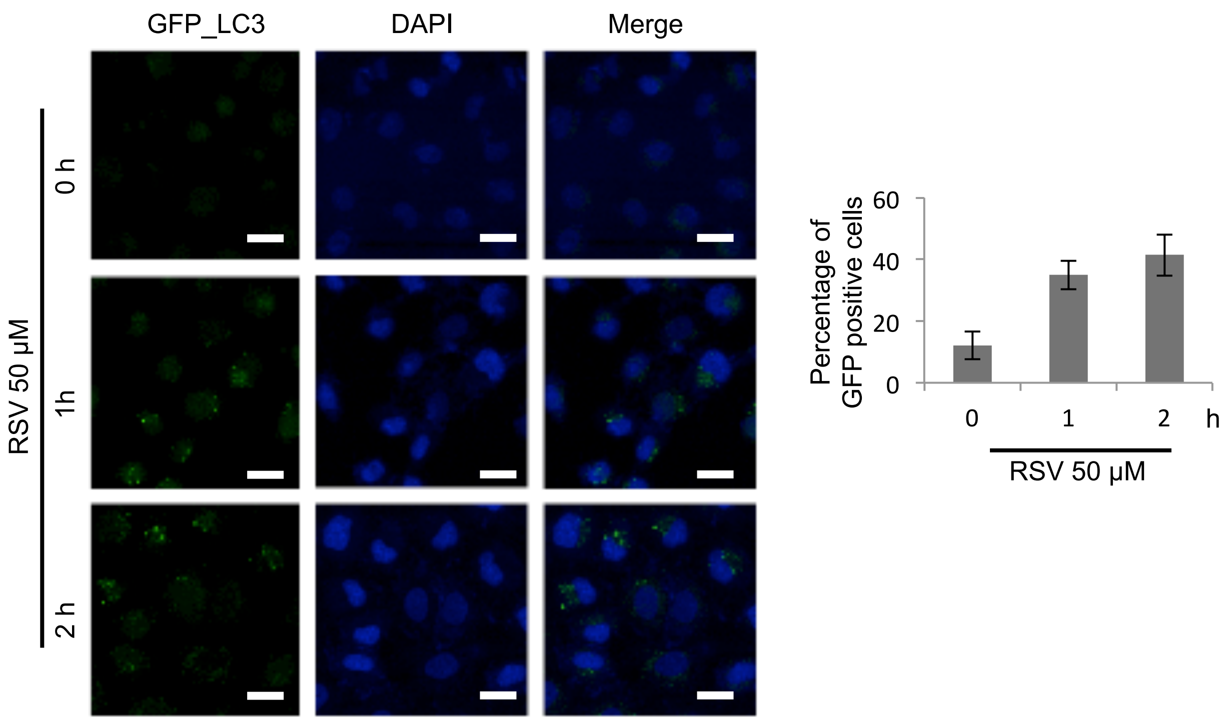

GFP_LC3
DAPI
Merge
0 h
1h
2 h
RSV 50 μM
Percentage of GFP positive cells
60
40
20
0
0
1
2
h
RSV 50 μM

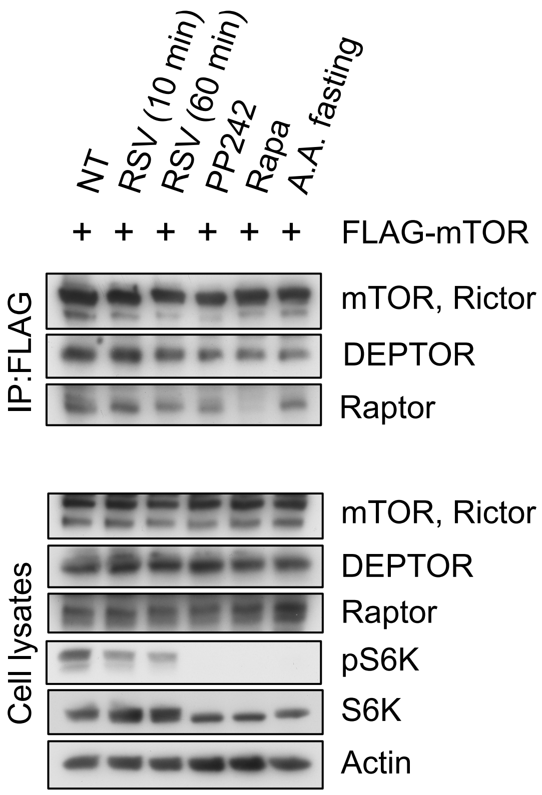
NT
RSV (10 min)
RSV (60 min)
PP242
Rapa
A.A. fasting
+ + + + + +
FLAG-mTOR
IP:FLAG
mTOR, Rictor
DEPTOR
Raptor
Cell lysates
mTOR, Rictor
DEPTOR
Raptor
pS6K
S6K
Actin

Figure S1. Resveratrol induces autophagy formation.

A. Resveratrol (50 μM) or PP242 (1.25 μM) was administered to HEK293 cells to measure the accumulation of LC3B-II.
B. LC3 puncta formation by resveratrol was measured. Resveratrol (50 μM) was administered to into GFP-LC3 expressing HeLa cells for the indicated times. Scale bars in fluorescent pictures represent 20 μm

Figure S2. DEPTOR affinity was not enhanced by resveratrol

The level of interaction between mTOR and DEPTOR, Raptor, and Rictor was assessed. FLAG-tagged mTOR was introduced into HEK293 cells. Chemical treatment (resveratrol: 50 μM, 10 min or 60 min; PP242: 1.35 μM 1h; rapamycin: 20 nM, 1h) or amino acid fasting (HBSS incubation for 30 min) was administered followed by immunoprecipitaion of mTOR using anti-FLAG antibody conjugated beads.

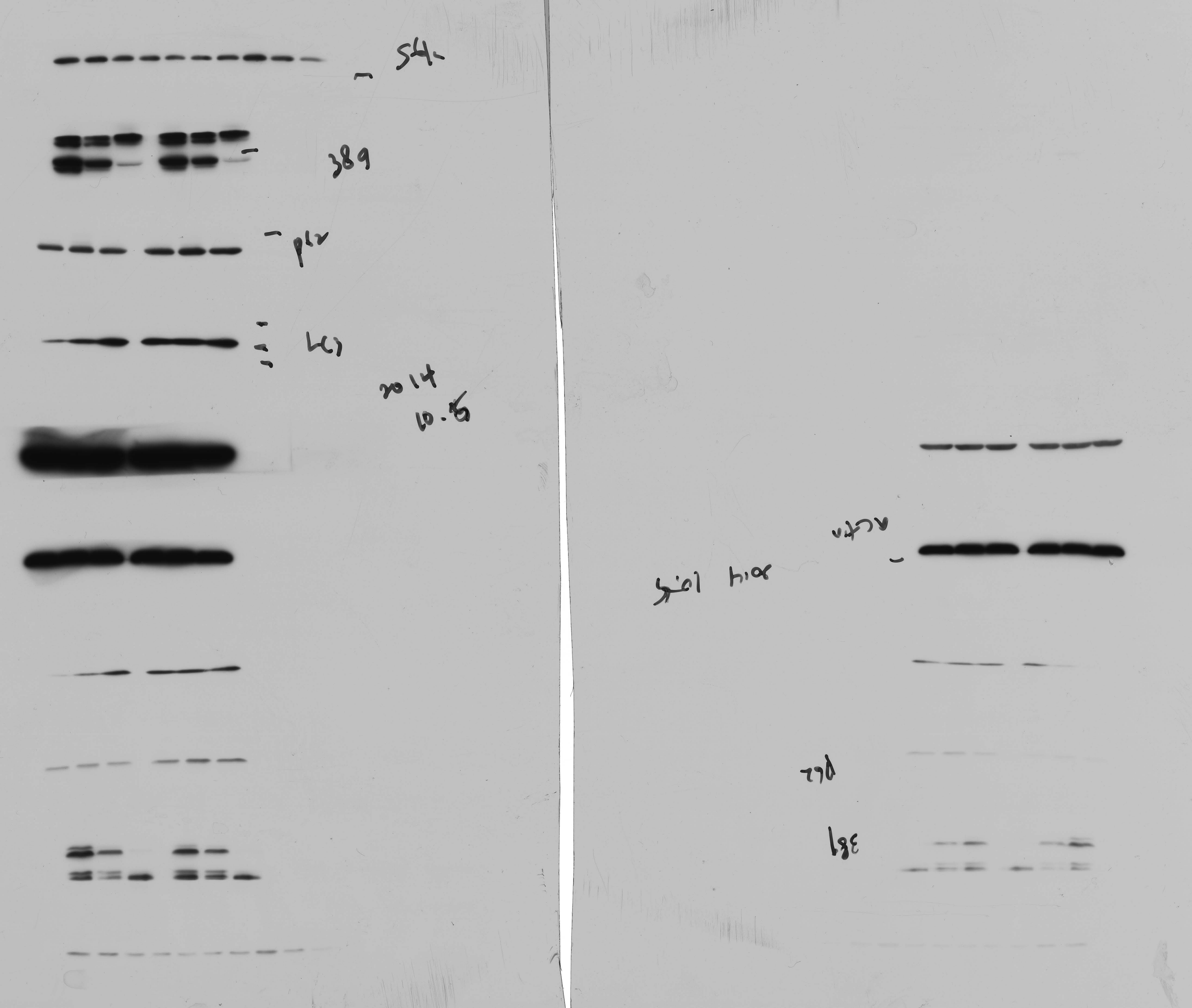
389
2014

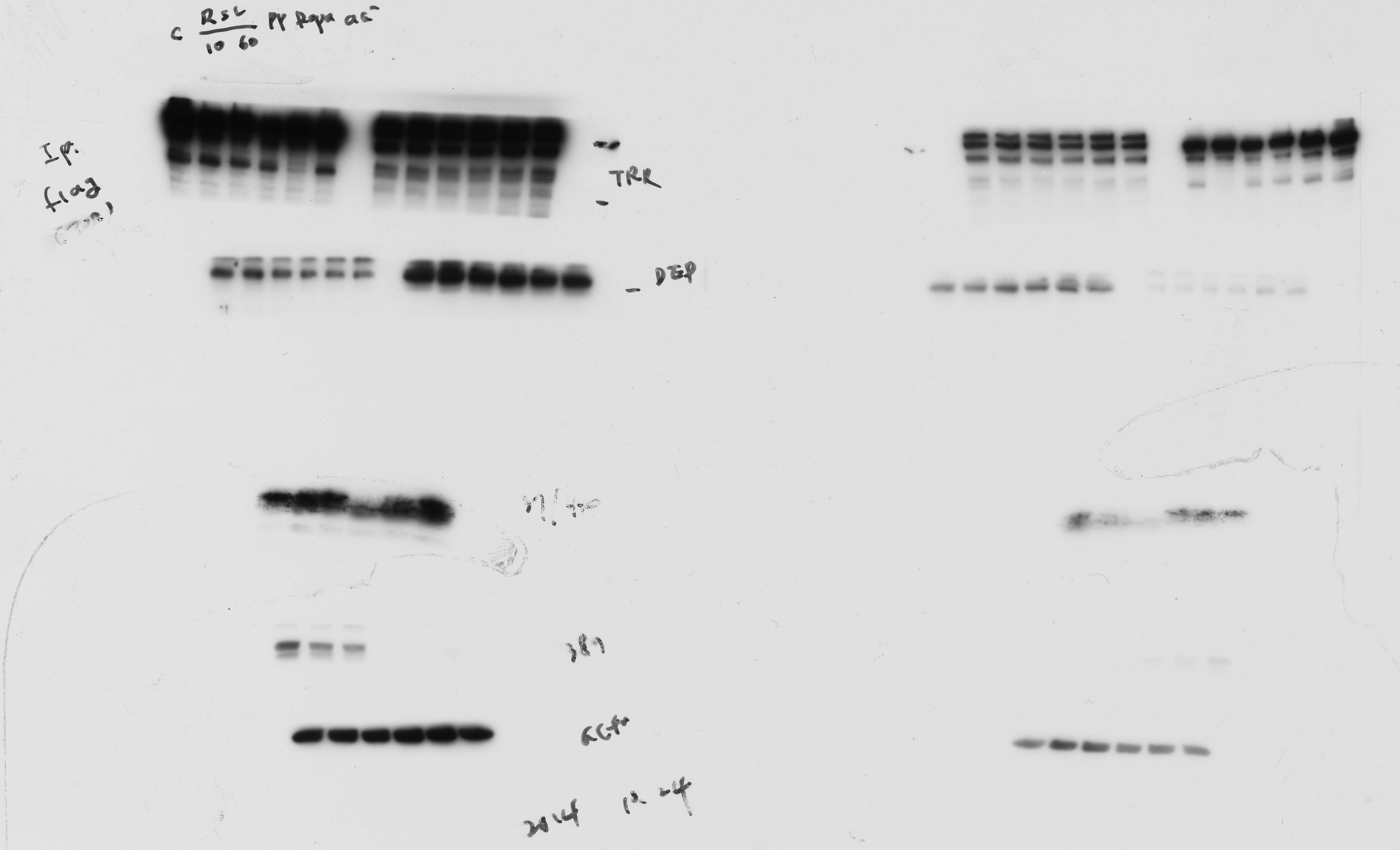
c
Rsl
10 60
Ip.
flag
TRR
DEP

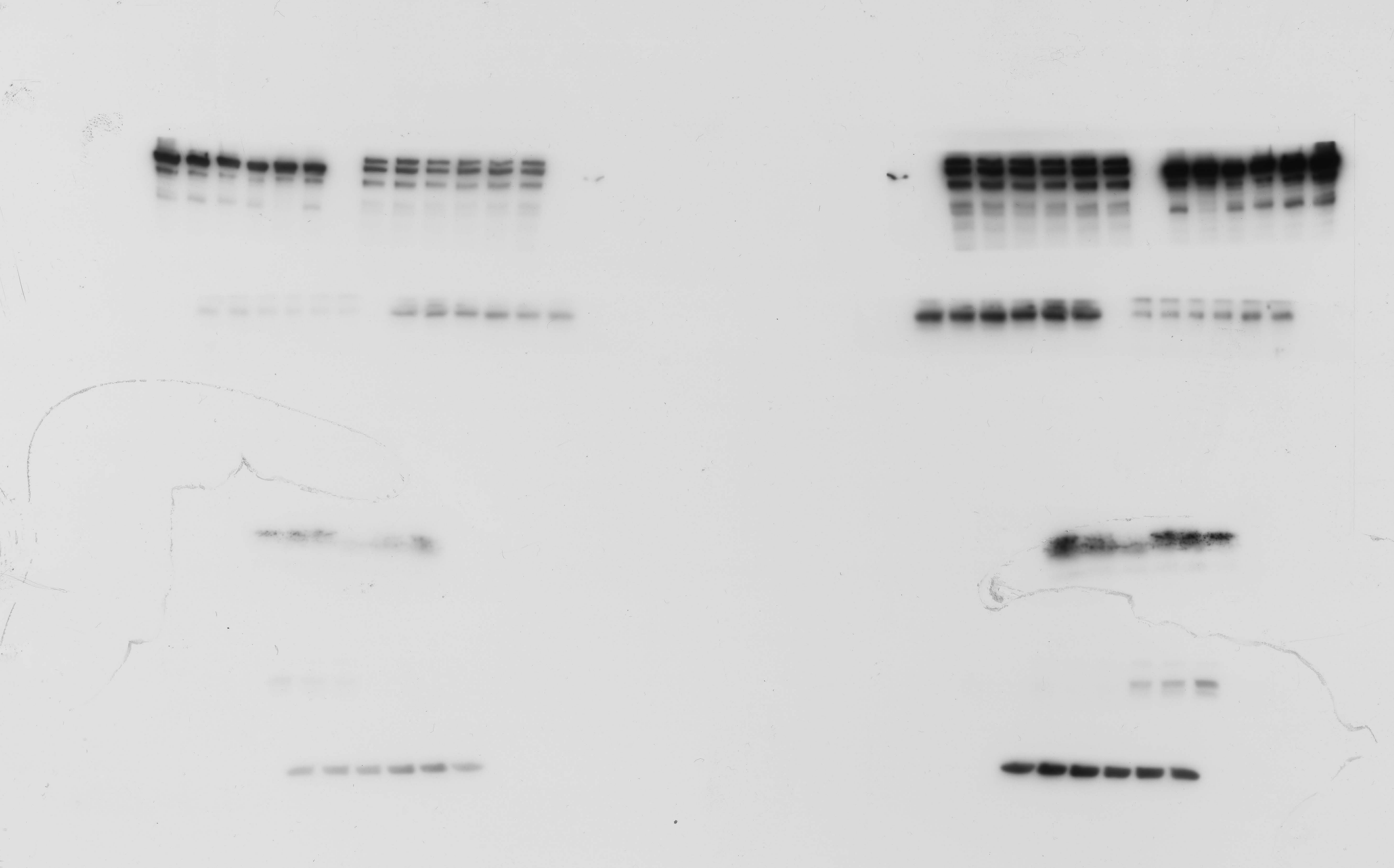

Supplement: Supplementary Information [file srep21772-s1.pdf]
